# Metastatic squamous cell carcinoma of mediastinal lymph nodes from the ileum: a case report and literature review

**DOI:** 10.3389/fonc.2025.1579029

**Published:** 2025-07-29

**Authors:** Yanan Zhang, Yumeng Yue, Zongxing Zhao, Haifeng Liu

**Affiliations:** ^1^ Department of Geriatrics, Liaocheng People’s Hospital, Liaocheng, Shandong, China; ^2^ Department of Radiation Oncology, Liaocheng People’s Hospital, Liaocheng, Shandong, China

**Keywords:** squamous cell carcinoma, mediastinal lymph nodes, ileum, case report, metastasis

## Abstract

Mediastinal lymph node metastasis from squamous cell carcinoma (SCC) is one of the most common sites for regional metastasis in thoracic malignancies. However, SCC originating from the ileum is relatively rare. We herein report a case of mediastinal lymph node metastasis as the first manifestation of postoperative ileal SCC. No primary tumor was detected on comprehensive imaging studies and bronchoscopy in this patient, and endobronchial ultrasound (E-BUS) considered metastatic SCC. After undergoing four cycles of postoperative chemotherapy with the XELOX regimen, the patient experienced rapid disease progression; however, a subsequent change to a treatment regimen of paclitaxel and cisplatin resulted in a favorable response. This article details the patient’s clinical presentation and treatment course and includes a literature review of primary SCCs arising from the jejunum and ileum, thereby enhancing understanding of these uncommon small intestine tumors.

## Introduction

During malignant tumor metastasis, lymph nodes are anatomically situated downstream of the tumor tissue and are regarded as gateways to distant metastasis. While mediastinal lymph node metastasis is more commonly associated with malignant tumors of the upper gastrointestinal tract, it is rarer in lower gastrointestinal tract cancers. Small bowel malignancies account for only 3% of gastrointestinal tumors, making them relatively uncommon (1). Most small bowel cancers develop in the duodenum, and SCC originating from the jejunum is extremely rare (2). Here, we present a case of SCC with mediastinal lymph node metastasis originating from the ileum, and summarize nine previously reported cases in the English literature.

## Case presentation

**Figure 1:**
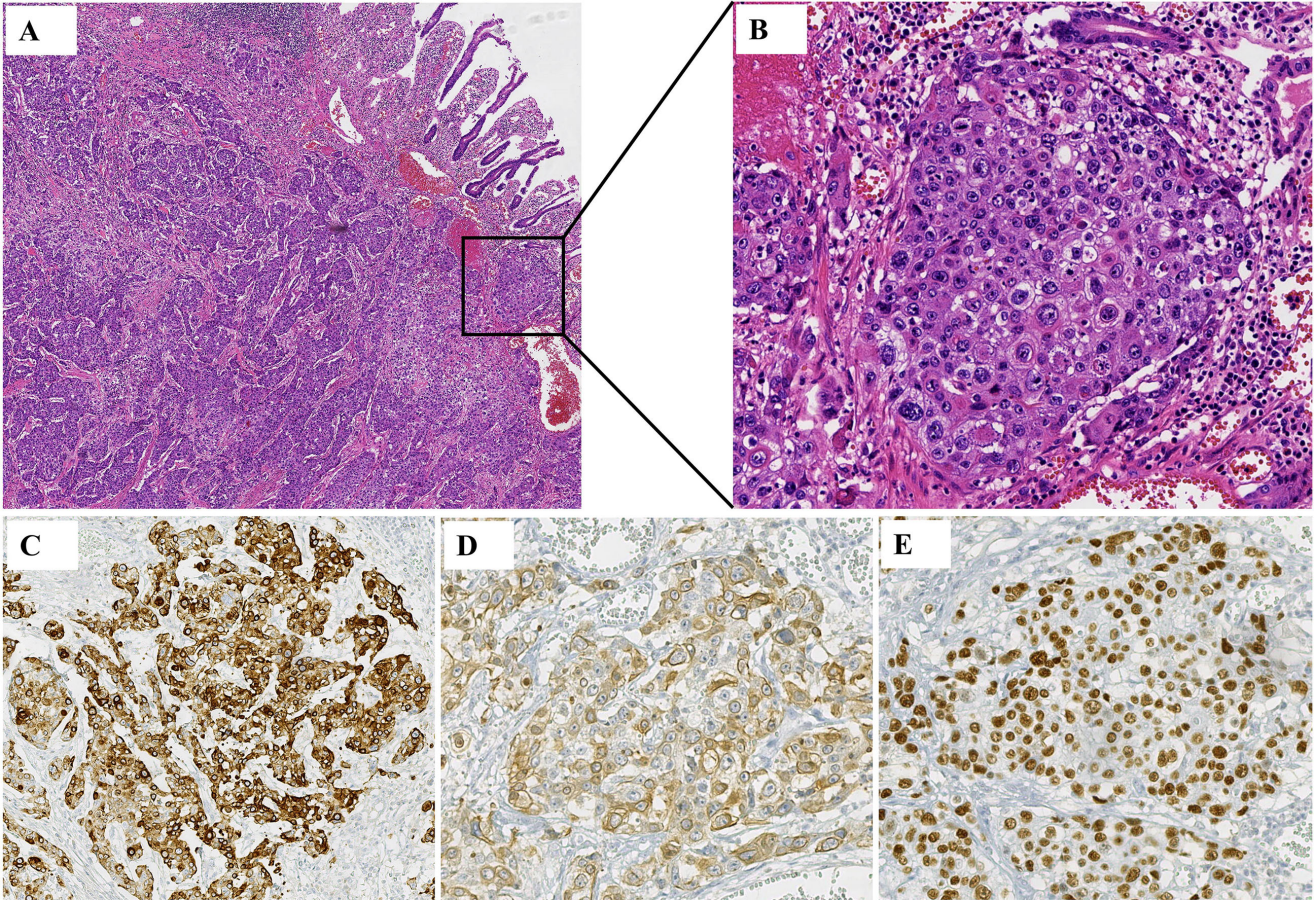
Histology from a postoperative ileum mass. **(A, B)** Squamous cell carcinoma was seen in the patient’s tissue. **(A)** HE, ×5; **(B)** HE, ×20. **(C)** Immunohistochemistry showed that CK was positive in the patient’s tissues. **(D)** Immunohistochemistry showed that CK5/6 was positive in the patient’s tissues. **(E)** Immunohistochemistry showed that P63 was positive in the patient’s tissues.

A 52-year-old male patient with a 20-year history of alcohol consumption presented to our gastrointestinal surgery department in July 2023, complaining of intermittent abdominal pain lasting ten days, which had intensified over the past day. The patient has no family history of malignancy. Upon examination, the patient exhibited abdominal muscle tension, pressure pain throughout the abdomen, a drum-like sound upon percussion, and reduced bowel sounds. An abdominal CT scan indicated abdominopelvic effusion and free gas within the peritoneal cavity. With a suspicion of digestive perforation, the patient underwent an exploratory laparotomy under general anesthesia following a comprehensive preoperative evaluation. During the surgery, a mass measuring 5x5x3 cm was discovered at the terminal ileum, approximately 5 cm from the ileocecal valve, along with an enlarged lymph node measuring about 4x4x3 cm at the base of the ileocecal blood vessels. Histological examination of the surgical specimen confirmed moderately differentiated ulcerative SCC with subserosal infiltration (pT3), lymphovascular invasion, and lymph node metastases (2/26). The immunoprofile supported squamous differentiation: CK(+), CK5/6(+), P63(+), P53(-), D2-40(+), HER-2(0), SMARCA4/Brg1(+), AFP(-), CgA(-), Syn(-), CD56(-), SALL-4(-) (**Figure 1**), with intact mismatch repair proteins (MLH1+, PMS2+, MSH2+, MSH6+). The proliferative index (Ki-67 at 95%) indicated a high-grade biological behavior. Postoperative supplemental imaging, including a chest-enhanced CT, cranial MRI, and bone scans, revealed no metastatic lesions. Given the rarity of small intestine SCC, postoperative adjuvant chemotherapy was administered using the XELOX regimen, following the NCCN guidelines for small intestine adenocarcinoma (3). After surgery, the patient underwent four cycles of chemotherapy with the XELOX regimen starting from August 16, 2023. The patient experienced mild fatigue and nausea as adverse effects during chemotherapy, which were assessed as grade 1 according to the CTCAE 5.0 criteria.

**Figure 2:**
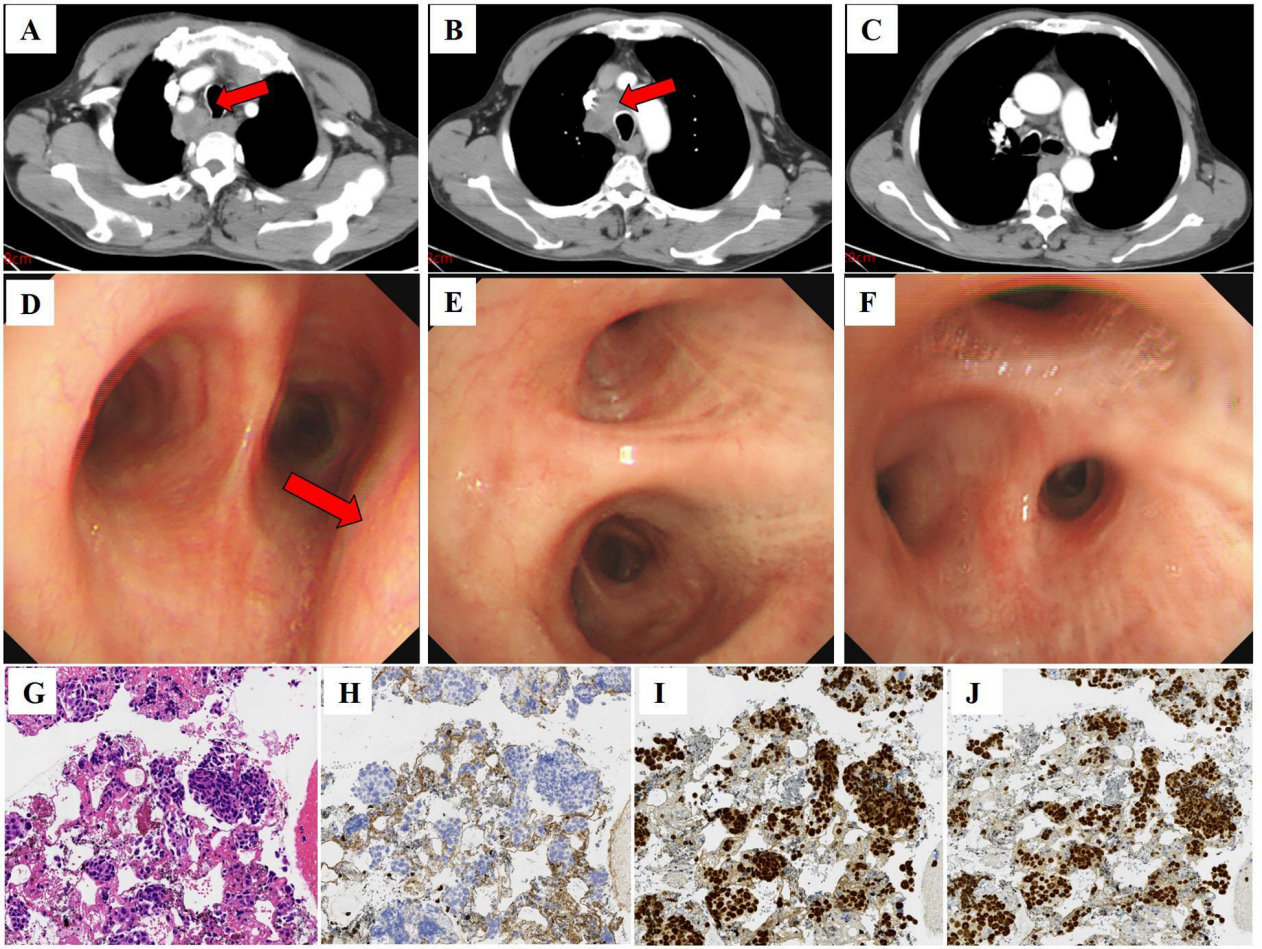
Computed tomography, bronchoscopy images, and histology of punctured tissue of the patient. **(A–C)** Computed tomography image shows enlarged lymph nodes in the mediastinum (red arrow) (January 04, 2024; slice thickness: 5 mm; window width: 360; window level: 60; arterial phase). **(D–F)** Bronchoscopy shows extrinsic compressive stenosis of the tracheal lumen (red arrow), with primary and segmental bronchi patency and no hemorrhage or neoplasm. **(G)** Squamous cell carcinoma was seen in the patient’s tissue. HE, ×20. **(H)** Immunohistochemistry showed that CK7 was negative in the patient’s tissues. **(I)** Immunohistochemistry showed that P40 was positive in the patient’s tissues. **(J)** Immunohistochemistry showed that P63 was positive in the patient’s tissues.

The patient was admitted to our hospital in January 2024 due to the detection of a 33*35 mm mediastinal mass (**Figures 2A–C**) on chest CT. The asymptomatic patient exhibited elevated tumor markers: CEA 5.65 ng/mL and SCC antigen 28.0 ng/mL. Diagnostic workup revealed no primary lesions, including contrast-enhanced abdominal CT and bronchoscopy (**Figures 2D–F**). The endobronchial ultrasound guided tranbronchial needle aspiration (EBUS-TBNA) histopathology demonstrated poorly differentiated SCC with an immunohistochemical profile: CK5/6(+), P63(+), P40(+), CK7(-), CK20(-) (**Figures 2G–J**).

**Figure 3:**
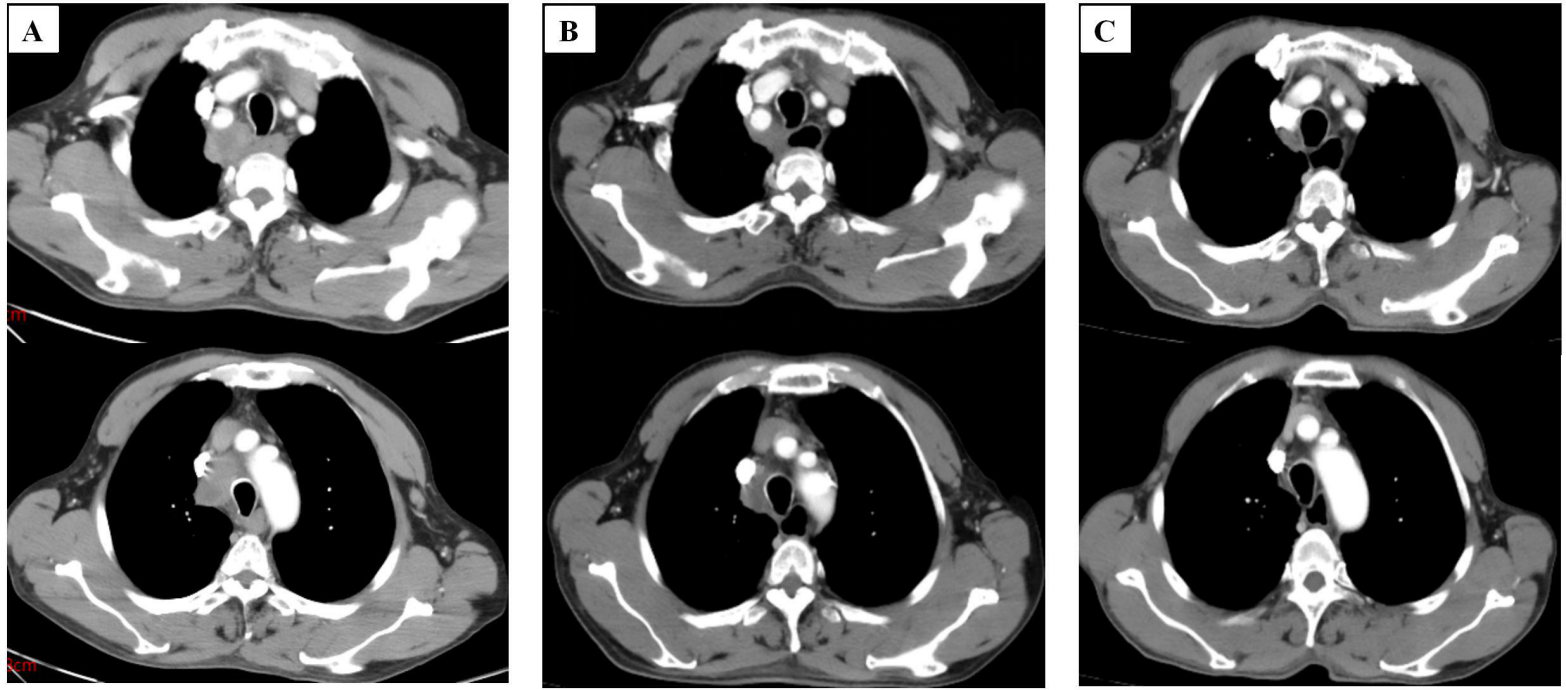
Computed tomography images of the patient. **(A)** Computed tomography image shows enlarged lymph nodes in the mediastinum before chemotherapy (January 04, 2024). **(B)** Computed tomography images of patients after 2 cycles of chemotherapy(February 23, 2024). **(C)** Computed tomography images of patients after 4 cycles of chemotherapy (April 8, 2024) (slice thickness: 5 mm; window width: 360; window level: 60; arterial phase).

**Figure 4:**
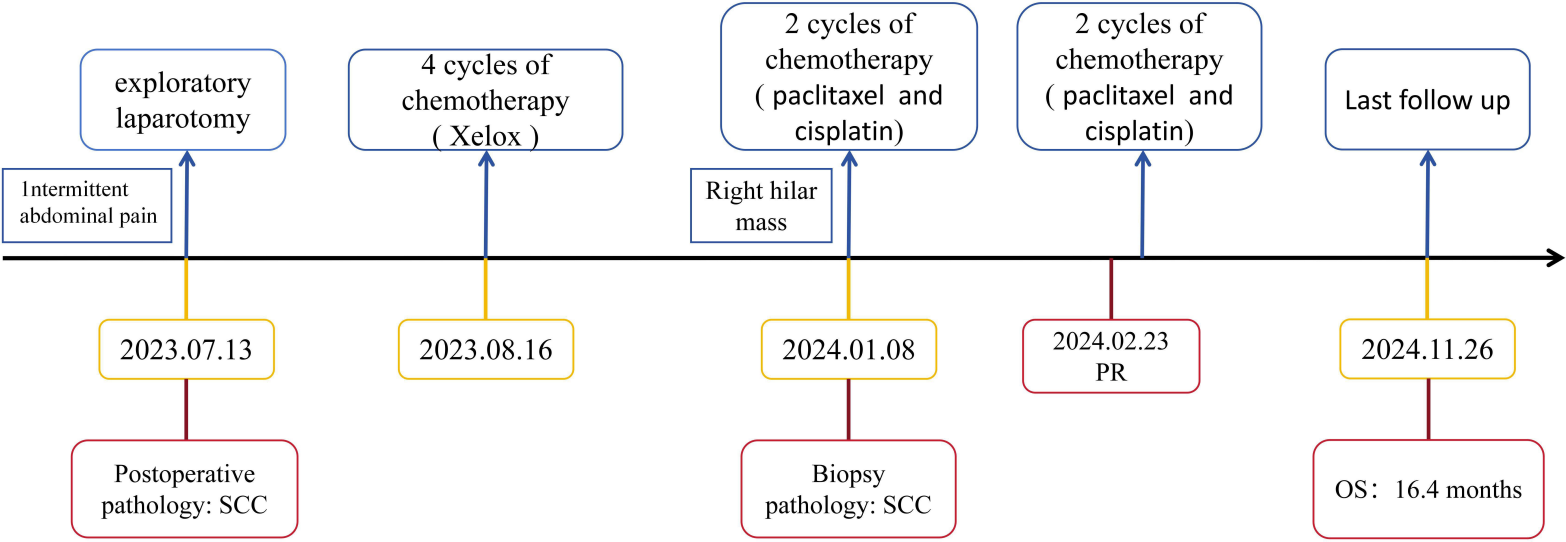
Timeline of the treatment. OS, overall survival; SCC, squamous cell carcinoma.

After considering the patient’s medical history, we attributed the lung hilar mass to metastatic squamous cell carcinoma (SCC) of ileal origin. Following four cycles of postoperative chemotherapy with the XELOX regimen, the patient’s condition progressed. Given the patient’s pathology, we sought a multidisciplinary team (MDT) discussion involving medical oncology, radiotherapy, thoracic surgery, pathology, imaging, and clinical nutrition. We adjusted the chemotherapy regimen to include paclitaxel (albumin-bound) (260 mg/m² on day 1 every 3 weeks) and cisplatin (80 mg/m² on day 1 every 3 weeks), starting on January 08, 2024. Throughout the chemotherapy, the patient experienced grade 1 fatigue and grade 2 bone marrow suppression, as defined by CTCAE 5.0, which both improved with symptomatic support. After two follow-up cycles, mediastinal lymph node shrinkage was observed. A Chest CT on February 23, 2024, revealed mediastinal lymph nodes measuring 18*21 mm (**Figure 3**). The treatment efficacy was assessed as partial remission according to Response Evaluation Criteria in Solid Tumors Version 1.1. After completing 4 cycles of chemotherapy, the patient underwent regular reviews. The last follow-up, on November 26, 2024, showed no significant progression or new metastatic lesions on chest CT. The patient’s overall survival stands at 16.4 months. **Figure 4** illustrates the patient’s entire treatment course.

## Discussion

This case highlights two salient clinical observations: (1) The exceptional metastatic pattern of ileal SCC to mediastinal lymph nodes, and (2) Differential chemosensitivity between fluoropyrimidine/oxaliplatin and taxane/platinum regimens in SCC histology.

Small bowel cancers are relatively rare compared to tumors in other gastrointestinal organs. They comprise a heterogeneous group of approximately 40 histological subtypes, the most common being adenocarcinomas, neuroendocrine tumors, mesenchymal tumors, and lymphomas, with SCCs exceptionally uncommon (2). In this case, HE staining confirmed SCC, and immunohistochemical analysis (CK7(-)) ruled out adenocarcinoma, while CK5/6(+), P63(+), and P40(+) supported the diagnosis of SCC. Small bowel SCC pathogenesis remains enigmatic, with proposed mechanisms including squamous metaplasia of adenomatous tissue or ectopic squamous epithelium (4). Our literature synthesis identified nine jejunoileal SCC cases (5–13) (**Table 1**), demonstrating male predominance (6:4) and variable survival (1–55 months). Notably, only two cases exhibited nodal metastasis, underscoring our patient’s unique dissemination pattern.

**Table 1:** Clinicopathologic features of primary jejunal and ileal squamous cell carcinoma reported in the literature.

Number	Author/year	Age/sex	Country	Stage	OS (months)	Location	Clinical presentation	Metastasis	Treatment
1	Sun DS et al. (5)/2014	80/male	Korea	pT3N0M0	NR	jejunum	abdominal cramps		surgery
2	Platt CC et al. (6)/1992	62/male	Japan	pT4N0M0	36	terminal ileum	abdominal colic and vomiting		surgery
3	Mumtaz S et al. (7)/2011	65/female	Pakistan	pT3N0M0	NR	ileum	abdominal pain and cramps, abdominal distension and blood in stools		surgery
4	Bao Y et al. (8)/2014	68/male	China	pT3N0M0	15	ileum	abdominal mass		surgery
5	Viamonte M et al. (9)/1992	65/female	USA	pT4N0M0	48	terminal ileum	abdominal pain, melena, and hematochezia		surgery
6	Xiao L et al. (10)/2022	69/male	China	NR	1	jejunum	abdominal pain	liver	surgery
7	Mino K et al. (11)/2012	72/male	Japan	pT4N1M0	55	ileum	appetite loss and abdominal distension	lymph nodes	surgery+ chemotherapy
8	Singh D et al. (12)/2023	55/female	India	pT4N0M0	NR	jejunum	abdominal pain		surgery
9	Cheng X et al. (13)/2009	59/male	China	pT2N0M0	NR	jejunum	abdominal pain		surgery

OS, overall survival; NR, not reported.

Currently, only nine cases of colorectal cancer metastasizing solely to mediastinal lymph nodes without lung involvement have been reported in the English literature (14). The paradoxical mediastinal metastasis from lower abdominal malignancies challenges conventional lymphatic drainage paradigms. Proposed mechanisms include:1. Thoracic duct reflux secondary to lymphatic valve incompetence from tumor obstruction; 2. Aberrant trans diaphragmatic lymphatic connections; 3. Hematogenous seeding with nodal tropism. The El-Halabi et al.’s colorectal cancer case (15) with isolated mediastinal involvement parallels our observation, suggesting potential shared pathways in the retroperitoneal-to-mediastinal spread.

Therapeutic optimization for small bowel SCC remains undefined due to rarity. While NCCN guidelines recommend XELOX for intestinal adenocarcinomas, our experience and Mino et al.’s report (16) suggest superior SCC responsiveness to paclitaxel/cisplatin regimens. The paclitaxel/cisplatin is the most important component of lung SCC, esophageal SCC, and head and neck SCC recommended chemotherapy regimens in the NCCN guidelines (17–19). This highlights the imperative for histology-driven therapy in gastrointestinal malignancies.

## Conclusion

This case expands the clinicopathological spectrum of small bowel SCC, emphasizing the diagnostic challenge of atypical metastatic presentations, the critical role of immunohistochemical profiling in tumor characterization, and the potential superiority of taxane-based regimens in SCC management. Further molecular characterization is warranted to establish evidence-based protocols for this orphan disease.

## Data Availability

The original contributions presented in the study are included in the article/supplementary material. Further inquiries can be directed to the corresponding author.

## References

[1] Siegel RL Miller KD Fuchs HE Jemal A. Cancer statistics, 2022. CA Cancer J Clin. (2022) 72:7–33. doi: 10.3322/caac.21708, PMID: 35020204

[2] Vlachou E Koffas A Toumpanakis C Keuchel M. Updates in the diagnosis and management of small-bowel tumors. Best Pract Res Clin Gastroenterol. (2023) 64-65:101860. doi: 10.1016/j.bpg.2023.101860, PMID: 37652650

[3] Chiorean EG Chiaro MD Tempero MA Malafa MP Benson AB Cardin DB. Ampullary adenocarcinoma, version 1.2023, NCCN Clinical Practice Guidelines in Oncology. J Natl Compr Canc Netw. (2023) 21(7):753–82. doi: 10.6004/jnccn.2023.0034, PMID: 37433437

[4] Wang FD Wang ZW Xue HD Wu HW Zhang Y Yu JC. Primary squamous cell carcinoma of the small intestine: pathogenesis and clinical features. Chin Med J (Engl). (2016) 129(17):2131–3. doi: 10.4103/0366-6999.189067, PMID: 27569244 PMC5009601

[5] Sun DS Shin OR Ku YM Kim YS Seo KJ. Squamous cell carcinoma of the small bowel manifesting as a jejunal perforation: a case report. Int J Clin Exp Pathol. (2014) 7:6345–9. doi: 10.23880/mjccs-16000354, PMID: 25337289 PMC4203260

[6] Platt CC Haboubi NY Schofield PF. Primary squamous cell carcinoma of the terminal ileum. J Clin Pathol. (1991) 44:253–4. doi: 10.1136/jcp.44.3.253, PMID: 2013630 PMC496951

[7] Mumtaz S Ahmad Z Fatima S Qureshi A. Squamous cell carcinoma in the small intestine. BMJ Case Rep. (2011) 2011:bcr0120113762. doi: 10.1136/bcr.01.2011.3762, PMID: 22696720 PMC3094779

[8] Bao Y Zhong ZX Yu YW. Squamous cell carcinoma of small intestine: a case report. Chin Med Sci J. (2014) 29:239–41. doi: 10.1016/s1001-9294(14)60078-x, PMID: 25429750

[9] Viamonte M Viamonte M. Primary squamous-cell carcinoma of the small bowel. Report of a case. Dis Colon Rectum. (1992) 35:806–9. doi: 10.1007/BF02050334, PMID: 1644008

[10] Xiao L Sun L Zhang JX Pan YS. Rare squamous cell carcinoma of the jejunum causing perforated peritonitis: A case report. World J Gastrointest Oncol. (2022) 14:2295–301. doi: 10.4251/wjgo.v14.i11.2295, PMID: 36438705 PMC9694277

[11] Mino K Kamii N Kawanishi N Okada T Todo S. Recurrence of primary squamous cell carcinoma of the ileum diagnosed by elevation of serum SCC: report of a case. Clin J Gastroenterol. (2012) 5:239–44. doi: 10.1007/s12328-012-0309-2, PMID: 26182328

[12] Singh D Kumar A Gowda V Sahai R Kumar S Kishore S. An adding up of an exceptionally rare case report: Primary Squamous cell carcinoma of Jejunum from North India. J Cancer Res Ther. (2023) 19:S451–3. doi: 10.4103/jcrt.JCRT_1333_20, PMID: 37148011

[13] Cheng X Wang ZG. Primary squamous carcinoma of intestine: report of a case. Zhonghua Bing Li Xue Za Zhi. (2009) 38:350–1. doi: 10.3760/cma.j.issn.0529-5807.2009.05.018 19575885

[14] Watanabe Y Suzuki R Kinoshita M Hirota M. Solitary anterior mediastinal lymph node metastasis with pericardial invasion from colon cancer: A case report. Mol Clin Oncol. (2022) 17:128. doi: 10.3892/mco.2022.2561, PMID: 35832473 PMC9264321

[15] El-Halabi MM Chaaban SA Meouchy J Page S Salyers WJ Jr. Colon cancer metastasis to mediastinal lymph nodes without liver or lung involvement: A case report. Oncol Lett. (2014) 8:2221–4. doi: 10.3892/ol.2014.2426, PMID: 25289100 PMC4186498

[16] Benson AB Venook AP Al-Hawary MM Arain MA Chen YJ Ciombor KK. Small bowel adenocarcinoma, version 1.2020, NCCN Clinical Practice Guidelines in Oncology. J Natl Compr Canc Netw. (2019) 17(9):1109–33. doi: 10.6004/jnccn.2019.0043, PMID: 31487687 PMC10191182

[17] Riely GJ Wood DE Ettinger DS Aisner DL Akerley W Bauman JR. Non-small cell lung cancer, version 4.2024, NCCN Clinical Practice Guidelines in Oncology. J Natl Compr Canc Netw. (2024) 22(4):249–74. doi: 10.6004/jnccn.2204.0023, PMID: 38754467

[18] Ajani JA D’Amico TA Bentrem DJ Cooke D Corvera C Das P. Esophageal and esophagogastric junction cancers, version 2.2023, NCCN Clinical Practice Guidelines in Oncology. J Natl Compr Canc Netw. (2023) 21(4):393–422. doi: 10.6004/jnccn.2023.0019, PMID: 37015332

[19] Caudell JJ Gillison ML Maghami E Spencer S Pfister DG Adkins D. NCCN guidelines^®^ Insights: head and neck cancers, version 1.2022. J Natl Compr Canc Netw. (2022) 20(3):224–34. doi: 10.6004/jnccn.2022.0016, PMID: 35276673

